# Exposure characteristics of familial cases of lung injury associated with the use of humidifier disinfectants

**DOI:** 10.1186/1476-069X-13-70

**Published:** 2014-09-02

**Authors:** Donguk Park, Jonghan Leem, Kyoungmu Lee, Heungkyu Lim, Yeyong Choi, Jong-Ju Ahn, Sinye Lim, Jeongim Park, Kyungho Choi, Naroo Lee, Hyejung Jung, Jongsik Ha, Domyung Paek

**Affiliations:** Department of Environmental Health, Korea National Open University, 169 Dongsung-dong, Jongno-gu, Seoul, 110-791 Republic of Korea; Department of Occupational & Environmental Medicine, Inha University Hospital, Incheon, Korea; Asian Citizen’s Center for Environment and Health, Seoul, 110-460 Korea; Department of Occupational & Environmental Medicine, College of Medicine, Kyunghee University, 130-791 Seoul, Korea; Department of Environmental Health Science, Soon Chun Hyang University, Chuncheongnam-do, 336-745 Korea; School of Public Health, Seoul National University, Seoul, 151-742 Korea; Occupational Safety and Health Research Institute, Korea Occupational Safety and Health Agency, Expo Yuseonggu, Daejun, 305-380 Korea; Korea Environment Institute, Seoul, 122-706 Korea

## Abstract

**Background:**

This study describes 17 families with 38 lung injury patients (14 males, 24 females; 22 preschool-age children less than six years of age and 16 individuals of 13–50 years) who used disinfectant added to humidifiers in the home.

**Methods:**

Clinical examination and humidifier disinfectant-use histories were taken, and a thorough home investigation was performed to assess exposure to humidifier disinfectant.

**Results:**

Nine of the patients (three pregnant females, six preschool-age children) died soon after they first developed lung damage. Six (16%) were pregnant females and 22 (58%) were preschool-aged children younger than six years. The patients used humidifier disinfectant products containing either polyhexamethylene guanidine phosphate (PHMG, *n* = 36) or oligo(2-(2-ethoxy)ethoxyethyl guanidinium chloride (PGH, *n* = 2). Twenty-six patients (68%) used the brand "Oxy"®, which contains PHMG. Of the ten patients with fatal lung injury, nine were found to have used PHMG.

**Conclusions:**

Our findings suggest that the use of humidifier disinfectant products containing either PGH or PHMG can cause lung injury, especially in preschool-age children younger than six years and pregnant women.

## Background

In South Korea, several types of disinfectants have been widely used in humidifiers since 1994 to prevent microbial contamination, but their use has been banned since 2011 due to concerns about their fatal health effects [1]. While disinfectant is used in other countries for humidifier cleaning [2], South Korea is believed to be the only country where a disinfectant was added to the water in humidifiers for extended periods of time. From August 2011 until recently, many related articles on the deaths of pregnant or postpartum females and infants who were exposed to humidifier disinfectant and developed interstitial lung disease of unknown causes made headlines in newspapers throughout South Korea. Several studies conducted in South Korea have concluded that humidifier disinfectants can cause fatal lung disease including interstitial pneumonitis and wide spread lung fibrosis [3–7]. To date, two hospital-based case–control studies have reported that the use of humidifier disinfectant was significantly associated with a cluster of interstitial lung disease (unadjusted OR = 47.3, 95% CI = 1.4-5.9) [6] (unadjusted OR = 2.7, 95% CI = 1.4-5.9) [7]. Those studies concluded interstitial lung disease were associated with the use of humidifier disinfectant. Microbiological results concluded that the possibility of an infectious disease was extremely low [3].

The objective of this study is to describe the characteristics of humidifier disinfectant exposures among self-reported lung injury patients.

## Methods

### Family lung injury cases

To address growing social concerns, Asian Citizen’s Center for Environment and Health (ACCEH), a Korean NGO, collected 174 cases of patients who voluntarily claimed that the use of humidifier disinfectant might have caused lung injuries to them from September 2011 to April 2012. Cases with either previous chronic respiratory diseases or other diseases that were not likely to be clinically related to the use of humidifier disinfectant were excluded. We, the Korea Society for Environmental Health (KSEH), revisited the cases registered with the ACCE Hand determined 95 lung injuries as being suspected of association with disinfectant use. Occupational physicians evaluated the clinical and radiological documentation they provided and the symptoms they reported [1]. Among all pulmonary diseases that may have been related to the use of humidifier disinfectant (n = 95), we selected 38 lung injury patients from the 17 families including a minimum of two lung injury patients in order to focus on cases with a greater possibility for the lung injury symptoms to have been caused by humidifier disinfectant.

Lung injury patients were classified into two categories according to the severity of lung damage: fatal and non-fatal lung injury. Both death and lung transplantation as a result of lung injury were classified as fatal injury. The criteria of diagnosis of the patients for humidifier disinfectant-related lung injury are as follows: Using humidifier disinfectant products in the home prior to the onset of the lung injury episodes; rapidly progressing interstitial lung injury after using humidifier disinfectant products; no interstitial lung injury prior to the use of humidifier disinfectant. Other reversible respiratory disease cases including interstitial pneumonitis, hypersensitivity pneumonitis, pneumonia and others were all categorized as non-fatal injury. We visited the 17 family homes with patients and conducted both personal interviews and field investigations.

### Exposure assessment

The following questionnaires were substantially used to assess the exposure to humidifier disinfectants in each household:

-  On average, how many months per year, how many weeks per month and how many days per week did you use humidifier disinfectant in the household?
-  As a daily average, how long did you spend in a room, including sleeping, where humidifier disinfectant was used?
-  What brands of humidifier disinfectant did you normally use?
-  When did you had 1^st^ lung injury symptoms?

A questionnaire was completed voluntarily by either the patient, their parents or guardians who agreed to participate in this investigation. We also investigatedwhether there was any exposure to chemical agents other than the humidifier disinfectant that might be related to the development of lung injury. Because detailed personal interviews and home investigations found no specific employment, lifestyle, or hazardous agents that could be suspected of having caused the lung disease, assessment results for those agents are not reported here. This study is a part of an investigation on the victims of humidifier disinfectants approved by institutional review board of School of Public Health, Seoul National University (42-2013-07-01).

### Data analysis

Demographic characteristics and humidifier disinfectant use-related information obtained from the questionnaire were categorized and described in accordance with the objectives of this study. A chi-square test was used to examine the association between the severity of lung injury dichotomized and demographics and humidifier disinfectant use characteristics. The duration of humidifier disinfectant use was categorized based on the distribution. All statistical analyses were performed using STATA ver. 12.

## Results

Nine deaths due to lung injury occurred among nine families. In one family, (F4) a pregnant woman received a lung transplantation and a four-year-old child died. All members of family F8 developed lung injury (Table 1).

**Table 1:** **Families with at least two lung injury patients**

Family ID	No. of family members	No. of patients	Type of lung injury, no.		
			Death	Lung transplantation	Non-fatal
F1	4	2	1		1
F2	4	3			3
F3	3	2			2
F4	4	2	1	1	
F5	4	2	1		1
F6	3	2			2
F7	5	4	1		3
F8	3	3			3
F9	4	2	1		1
F10	4	2			2
F11	3	2	1		1
F12	4	2			2
F13	3	2			2
F14	3	2	1		1
F15	3	2	1		1
F16	3	2			2
F17	4	2	1		1
Total	61^*^	38	9	1	28

^*^The family members not registered as victims (n = 23) + lung injury cases (n = 38) in the 17 study families.

The patients included six pregnant females and 22 preschool children younger than six years, accounting for 74% of all patients. The nine deaths due to lung injury were all among preschool children (*n* = 6) or pregnant females (*n* = 3) (Table 2). Three of the adult males who were office workers (F3, F8, F13) did not have a fatal lung injury.

**Figure 1:**
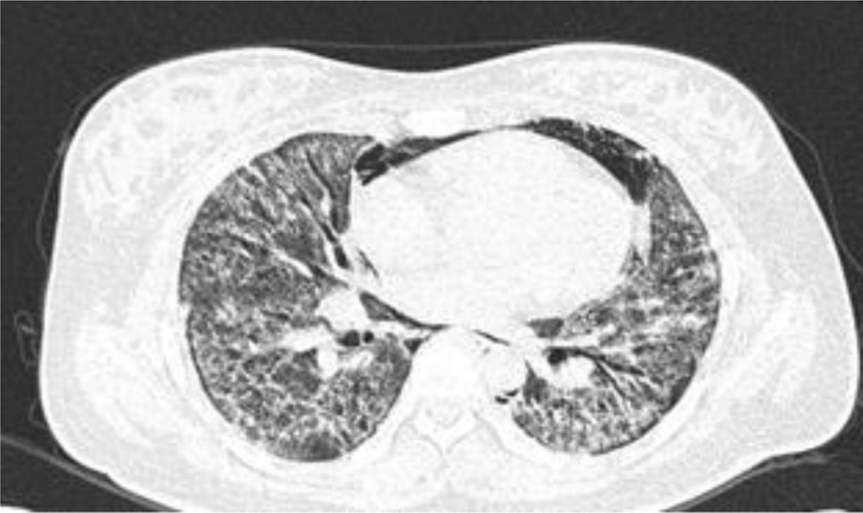
**Chest computed tomography (CT) image showing pneumomediastinum and bilateral diffuse ground glass opacity (GGO) lung infiltration in the peribronchial area in both lower lung fields in the initial phase observed in a fatal case (28 years old and pregnant female).**

We indicated a typical chest computed tomography (CT) image of a patchy nodular bilateral lung infiltration in the peribronchial area in both lower lung fields in the initial phase that was observed in a death due to fatal lung injury (Figure 1). Most of the non-fatal lung injuries showed clinical progression with acute interstitial pneumonia and interstitial fibrosis, leading to localized or reversible respiratory disease. Radiologic findings showed lung infiltration or lung consolidation, interstitial fibrosis, compatible with interstitial pneumonitis, pneumonia, and interstitial fibrosis.

**Table 2:** **Classification of lung injury patients according to demographics and lifestyle**

Classification	Total	Fatal	Non-fatal	***P-value***
Sex				
Male	14 (37%)	4 (40%)	10 (36%)	0.810
Female	24 (63%)	6 (60%)	18 (64%)	
Age				
≤6	22 (58%)	6 (60%)	16 (57%)	0.875
13-50	16 (42%)^#^	4 (40%)	11 (39%)	
Pregnant				
Yes	6 (16%)	4 (40%)	1 (4%)	NA
Smoking status				
Never	36 (95%)	10 (100%)	26 (93%)	0.262
Former	2 (5%)		2 (7%)	
Smoker in family				
Yes	20 (53%)	5 (50%)	15 (54%)	0.846
No	18 (47%)	5 (50%)	13 (46%)	
Current status				
Preschool child	22 (58%)	6 (67%)	12 (43%)	0.433
Student	1 (3%)		1 (4%)	
Housewife^#^	12 (32%)	4 (40%)	8 (29%)	
Office worker	3 (8%)	-	3 (11%)	

^#^Includes six pregnant women.

We confirmed the likelihood that a study subject used humidifier disinfectant based on both material evidence such as receipts for the purchase of disinfectant, the residual disinfectant, a photograph from the past showing the use of disinfectant and on consistency in statements regarding the use of disinfectant. The use characteristics of humidifier disinfectant are classified according to the severity of lung injury (Table 3). All patients responded that they used humidifier disinfectant before the onset of lung injury symptoms. Fatal cases used humidifier disinfectant for fewer total months compared to non-fatal cases (70% vs. 29%, <= 12 months), while other humidifier disinfectant use durations such as hours per day or month either after or before first onset are similar.

**Table 3:** **Classification of lung injury according to the use of characteristics of humidifier disinfectant**

Classification	Total	Fatal	Non-fatal	***P-value***
Use of humidifier disinfectant prior to the onset of 1^st^ lung injury				
Yes	38 (100%)	10 (100%)	28 (100%)	NA
No	0	0	0	
Total months of humidifier use				
<12	15 (37%)	7 (70%)	8 (29%)	0.011
12-24	13 (34%)	3 (30%)	10 (36%)	
30-72	11 (29%)		10 (36%)	
Sleeping in a room with a humidifier using disinfectant				
Yes	26 (68%)	7 (70%)	19 (68%)	0.900
No	12 (32%)	3 (30%)	9 (32%)	
Hours of humidifier disinfectant use per day*				
<11.5	19 (50%)	5 (50%)	14 (50%)	1.000
> = 11.5	19 (50%)	5 (50%)	14 (50%)	
Duration of humidifier disinfectant use after lung injury symptom onset, months				
<2	14 (37%)	5 (50%)	9 (32%)	0.320
> = 2	24 (63%)	5 (50%)	19 (68%)	
Duration of humidifier disinfectant use before lung injury symptom onset, years				
≤ 1	17 (45%)	7 (70%)	10 (36%)	0.060
1.1-19	21 (55%)	3 (30%)	18 (64%)	
Type of disinfectant				
PHMG	36 (95%)	9 (90%)	27 (96%)	0.462
PHG	2 (5%)	1 (10%)	1 (4%)	
Type of humidifier disinfectant				
Oxy®	26 (68%)	7 (70%)	19 (68%)	0.900
Other six brands	12 (32%)	3 (30%)	9 (32%)	

^*^Includes time sleeping,

*Abbreviations:*

PHMG = polyhexamethylene guanidine phosphate.

PGH = oligo(2-(2-ethoxy)ethoxyethyl guanidinium.

NA = not applicable.

All families used one for at least ten hours per day, including while sleeping. 7(70%) of fatal lung injury patients (n = 10) slept in rooms where humidifier disinfectant were used. All of the patients used either polyhexamethylene guanidine phosphate (PHMG, n = 36) or oligo(2-(2-ethoxy)ethoxyethyl guanidinium (PGH, n = 2). For 26 patients (68%) the humidifier disinfectant brand used was Oxy®, which contains PHMG. Seven of the death cases used this brand. PHMG was found to be the most commonly used humidifier disinfectant. No inhalation toxicological information was provided in the relevant material safety data sheets.

## Discussion

More than 10 humidifier disinfectant products containing several types of disinfecting compounds (PHMG, PGH, CMIT, MIT, and sodium dichloro-s-trianinetroione) had been marketed in South Korea. There were no test requirements for toxicological effects or inhalation health effects prior to market, until November 2011 when six brands containing PHMG and PGH were banned [1].

Since the identification of humidifier disinfectant being associated with lung injury, two toxicological studies were conducted to examine the toxicological effect of PHMG and PGH, the major components of humidifier disinfectant in South Korea. In a histopathological study using rats, the findings for products containing PHMG and PGH were identical to those regarding the patients with lung injury [8]. Kim et al. observed granulomatous obliterative bronchiolitis (OB), bronchitis, collagenized fibrosis, alveolar bronchiolarization, and extensive squamous metaplasia in rats exposed to humidifier products with PHMG and PGH at concentrations of 0.4 mg/m^3^ and 1.75 mg/m^3^ for ten and seven weeks, respectively [9]. We estimated airborne PHMG level based on PHMG level of disinfectant dissolved in humidifier disinfectant product (range: 1,276 to 6,917 ug/mL) reported by the Korea Centers for Disease Control and Prevention (KCDC) to a 2013 parliamentary [10]. If 10 mL of a humidifier disinfectant product featuring PHMG with 6,917 μg/mL (0.6917%) were used for one day and dispersed completely into a room with a volume of 27 m^3^ (a typical room size in South Korea), the airborne PHMG concentration could be approximately 2.56 mg/m^3^. This estimated concentration is similar to the level (2.75 mg/m^3^) of polyhexamethylene biguanidine (PHMB) at which all animals in an experiment died by Day 7 with moderate to severe pneumonitis [11]. PHMB is a broad-spectrum antimicrobial agent that induces membrane damage and genomic alterations in microorganisms [12] and is a polymer of PHMG,

Our descriptive study of family lung injury cases supports the hypothesis that the use of disinfectant in humidifiers containing either PHMG or PGH is likely the major cause of the lung injury, especially in the preschool children younger than six years and in pregnant females, based on the following findings.

First, no lung injuries with clinical features similar to those of our cases have been reported since the banning of six humidifier disinfectant products containing either PHMG or PGH in South Korea in November 2011. Recently, this assumption was confirmed by Kim et al. [13] who concluded through a nationwide surveillance study in Korea assessing the clinical characteristics of 138 children’s interstitial lung diseases registered between 2006 and 2011, no more new cases had been found two years after the ban of humidifier disinfectant sales.

Second, all patients with fatal lung injuries or requiring lung transplantation were preschool-age children (n = 6) and pregnant females (n = 4), who spent more time within the household than other family members. This result is similar to high mortality including 80 deaths (58%) among 138 child interstitial lung disease patients in Korea who were reported to be associated with use of humidifier disinfectant [13]. Preschool-age children definitely develop higher exposure in the home environment than do other family members because they spend much of their time at home. Infants and young children are suspected to be at high risk since they take in more of a contaminant, such as a disinfectant, relative to their body size than do adults and have particularly vulnerable physiologies. All pregnant females responded that they had started using humidifier disinfectant because they used humidifiers throughout the winter when they were pregnant or after giving birth. Seven (70%) out of 10 fatal lung injury cases from the preschool-age children and pregnant women patient groups were found to have used humidifier disinfectant for less than 12 months (Table 3). These two patient groups were found to have used humidifier disinfectant intensively during a specific period of child development, such as in the pre-gestation, gestational, and post-natal periods. These periods may have caused increased susceptibility to lung injury. In particular, preschool-age children differ substantially from adults in terms of exposure (greater intake of air), physiological factors (greater circulatory flow rates, pharmacokinetics (respiratory absorption rates) and pharmacodynamics (immature host defenses) [14, 15], all of which may be closely associated with the high incidence rate of lung injury cases.

The 23 family members not registered as victims among the 17 study families were adults between the ages of 36 and 59 years old (15 males, five females) and two females aged 11 and 14 years (F12) who were likely far less exposed to humidifier disinfectant than were their patient family members (Table 1). In one study family, a healthy four-year-old child (F17) did not sleep in a room with a humidifier using disinfectant. However, we did not investigate both the characteristics of humidifier disinfectant use and respiratory symptoms of healthy family members.

Lastly, all patients recalled using humidifier disinfectant products including either PHMG or PGH at home prior to each episode of lung disease. The majority of patients (68%) from 12 families used the brand Oxy®, which contains PHMG. Seven of the nine deaths and one lung transplantation involved patients from families that had used this particular product (Table 3). Detailed personal interviews and home investigations found no specific employment, lifestyle, or hazardous agents that could be suspected of having been the cause of lung injury, especially fatal lung damage.

It is plausible for humidifier disinfectants to have been inhaled by the study subjects and potentially cause lung damage. The ultrasonic humidifiers used by most patients readily disperse aerosol water droplets ranging in size from 0.5 to 3 μm, which easily reach the distal airways [16, 17]. The mist containing disinfectant aerosolized during humidifier operation might be inhaled by individuals near the humidifier. Importantly, the KCDC reported that 30–80-nm nanoparticles of PHMG or PGH can be aerosolized when humidifier disinfectant are used in ultrasonic humidifiers [18]. This aerosolized humidifier disinfectant can subsequently be deposited in the respiratory system and might cause interstitial lung disease in children [7].

Due to increased social concern, as of September 2013 the KCDC has officially collected 374 lung injury cases, including those reported here, registered as lung injury cases due to the use of humidifier disinfectant. It has conducted a detailed investigation to determine if these cases are associated with the use of disinfectant. Our results will be useful for conducting clinical examinations and exposure assessments for the registered patients in order to better characterize the association between exposure to humidifier disinfectant and various types of lung injury. The strengths of this study are not only the assessment of several humidifier-disinfectant exposure characteristics of lung injury patients, but also identifying specific types of disinfectant such as PHMG and PGH associated with lung injury risk.

The major limitation of this descriptive study is that there could be a risk of recall bias, because most disinfectant use characteristics were obtained predominantly from direct reports from study subjects. We increased the accuracy of responses by showing a photograph of each disinfectant brand and asking additional questions that may be specific to respective disinfectant types. Lung injury patients who were evaluated to be associated with the use of humidifier disinfectant may have searched their humidifier disinfectant use histories for explanations, priming greater recall of causal agents This type of bias occurs when cases are aware of the study hypothesis, resulting in higher exposure reporting [19]. A control group should be invited in order to minimize this reporting bias. Based on this experience, a non-lung injury familial group who lived in the same residence as lung injury patient group is being used as a control group in our further study currently underway. In addition, we did not consider humidifier disinfectant use characteristics among fatal or non-fatal cases according to their mobility or the amount of time they spent in the home, which may cause a difference in humidifier disinfectant use characteristics between fatal and non-fatal cases (Table 3). A more refined exposure assessment approach than that which was used to estimate the probability, frequency, and intensity of exposure to humidifier disinfectants should be developed based on mobility and the amount of time spent in the home and the duration during which biocide was actually used.

Examining the mechanism of how humidifier disinfectant eventually results in interstitial lung disease is challenging because there is limited knowledge regarding the potential exposure levels experienced by different age groups of victims with a wide range of susceptibilities, as well as on the biological activity and toxicity of several disinfectant chemical constituents, both alone and in combination. Numerous studies are underway in Korea to clinically confirm lung injury patients who used humidifier disinfectant, evaluate the long-term health effect on the clinically confirmed lung injury patients, and associate the risk of interstitial lung disease with humidifier disinfectant-related use characteristics. Furthermore, a retrospective cohort study should be conducted in order to evaluate health effects, including lung injury, in the general population who used humidifier disinfectant at some point from 1994 until the ban of humidifier disinfectants in November 2011.

## Conclusions

The use of humidifier disinfectant products containing either PHMG or PGH at home has the potential to cause lung injury, especially in susceptible populations such as infants, preschool-age children, and pregnant women.

## Abbreviations

CMIT: Chloromethylisothiazolinone
PGH: Oligo(2-(2-ethoxy)ethoxyethyl guanidinium
PHMG: Polyhexamethylene guanidine phosphate
MIT: Methylisothiazolinone
KCDC: Korea Centers for Disease Control and Prevention.
